# Exosomes in brain diseases: Pathogenesis and therapeutic targets

**DOI:** 10.1002/mco2.287

**Published:** 2023-06-11

**Authors:** Qingying Si, Linlin Wu, Deshui Pang, Pei Jiang

**Affiliations:** ^1^ Department of Endocrinology Tengzhou Central People's Hospital Tengzhou China; ^2^ Department of Oncology Tengzhou Central People's Hospital Tengzhou China; ^3^ Translational Pharmaceutical Laboratory Jining First People's Hospital Shandong First Medical University Jining China; ^4^ Institute of Translational Pharmacy Jining Medical Research Academy Jining China

**Keywords:** biomarker, diagnosis, exosomes, neuropsychiatric diseases, treatment

## Abstract

Exosomes are extracellular vesicles with diameters of about 100 nm that are naturally secreted by cells into body fluids. They are derived from endosomes and are wrapped in lipid membranes. Exosomes are involved in intracellular metabolism and intercellular communication. They contain nucleic acids, proteins, lipids, and metabolites from the cell microenvironment and cytoplasm. The contents of exosomes can reflect their cells’ origin and allow the observation of tissue changes and cell states under disease conditions. Naturally derived exosomes have specific biomolecules that act as the “fingerprint” of the parent cells, and the contents changed under pathological conditions can be used as biomarkers for disease diagnosis. Exosomes have low immunogenicity, are small in size, and can cross the blood–brain barrier. These characteristics make exosomes unique as engineering carriers. They can incorporate therapeutic drugs and achieve targeted drug delivery. Exosomes as carriers for targeted disease therapy are still in their infancy, but exosome engineering provides a new perspective for cell‐free disease therapy. This review discussed exosomes and their relationship with the occurrence and treatment of some neuropsychiatric diseases. In addition, future applications of exosomes in the diagnosis and treatment of neuropsychiatric disorders were evaluated in this review.

## Graphical abstract

Exosomes from neural cells can cross the blood–brain barrier, which can be used for diagnosis. Meanwhile, exosomes can be modified and engineered by loading specific nucleic acids, proteins, and drugs, which can be peripherally injected and play a therapeutic role to restore the health of the brain.

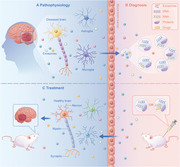

## 1 INTRODUCTION

Exosomes are extracellular vesicles (EVs) secreted by almost all cell types.^1^, ^2^, ^3^ They are formed by indenting the cell membrane, enveloping the extracellular microenvironment and cytoplasmic material, and exiting the cell before extracellular action.^4^, ^5^, ^6^, ^7^, ^8^, ^9^ Exosomes contain sugars, lipids, proteins, nucleic acids, and bioactive substances in the extracellular matrix,^10^ which was initially thought to be a cellular mechanism to eliminate metabolic waste.^11^ Recent studies have shown that released exosomes can be used by other cells to mediate intercellular communication and participate in the physiological and pathological processes of the body.^12^, ^13^, ^14^, ^15^ The function of exosomes depends on the type of cell origin.^16^ They are involved in the immune response,^15^, ^17^, ^18^, ^19^ antigen presentation, cell migration, differentiation and angiogenesis,^20^, ^21^ inflammatory induction,^22^ oxidative stress and apoptosis,^23^ atherosclerosis,^24^, ^25^ tumor initiation, progression, invasion and metastasis, and drug resistance.^26^, ^27^ Given the ability of exosomes to carry and transfer bioactive substances,^28^ the potential of exosomes to reveal the pathogenesis of diseases such as cancer,^29^, ^30^, ^31^, ^32^, ^33^ brain diseases,^34^, ^35^, ^36^, ^37^ cardiovascular diseases,^38^, ^39^, ^40^, ^41^, ^42^, ^43^, ^44^ and metabolic diseases,^45^, ^46^, ^47^ as well as to diagnose and treat diseases as biomarkers are being studied and explored.^48^, ^49^, ^50^, ^51^, ^52^

The prevalence of neuropsychiatric disorders, the leading cause of disability in humans, has increased in the last few decades^53^ and continues to grow due to the coronavirus disease (COVID‐19) outbreak in 2019.^54^, ^55^ Despite numerous studies, the exact etiology of neuropsychiatric disorders is still unclear. Diagnosis is based primarily on subjective cognitive evaluation. There are no widely recognized objective indicators or biomarkers for early diagnosis, limiting the effective diagnosis and treatment of neuropsychiatric disorders.^53^ Exosomes, as a new type of liquid biopsy, can potentially diagnose and predict the prognosis of diseases.^56^, ^57^, ^58^, ^59^ At the same time, exosomes are also high‐quality carriers of bioactive molecules or therapeutic drugs.^60^, ^61^, ^62^, ^63^, ^64^, ^65^, ^66^ Their modification can target the injury site for precise treatment.^67^, ^68^, ^69^, ^70^, ^71^, ^72^ The contents of exosomes undergo specific changes when the internal and external environment changes, causing tissue dysfunction and disease.^73^, ^74^, ^75^, ^76^, ^77^, ^78^ The detection and modification of exosomes provide a new perspective for diagnosing and treating neuropsychiatric diseases, and their appearance offers a fresh approach for managing neuropsychiatric disorders.

Exosomes are involved in the pathophysiological processes of neuropsychiatric disorders, but their role in different neuropsychiatric disorders is not fully understood.^79^, ^80^, ^81^, ^82^, ^83^ Therefore, this review explores the mechanism of the pathogenesis of exosomes and therapeutic targets in brain diseases. Finally, the problems and challenges facing exosomes in future diagnoses and treatments of neuropsychiatric disorders are discussed.

## 2 EXOSOMES

### 2.1 Discovery and the structure of exosomes

**FIGURE 1:**
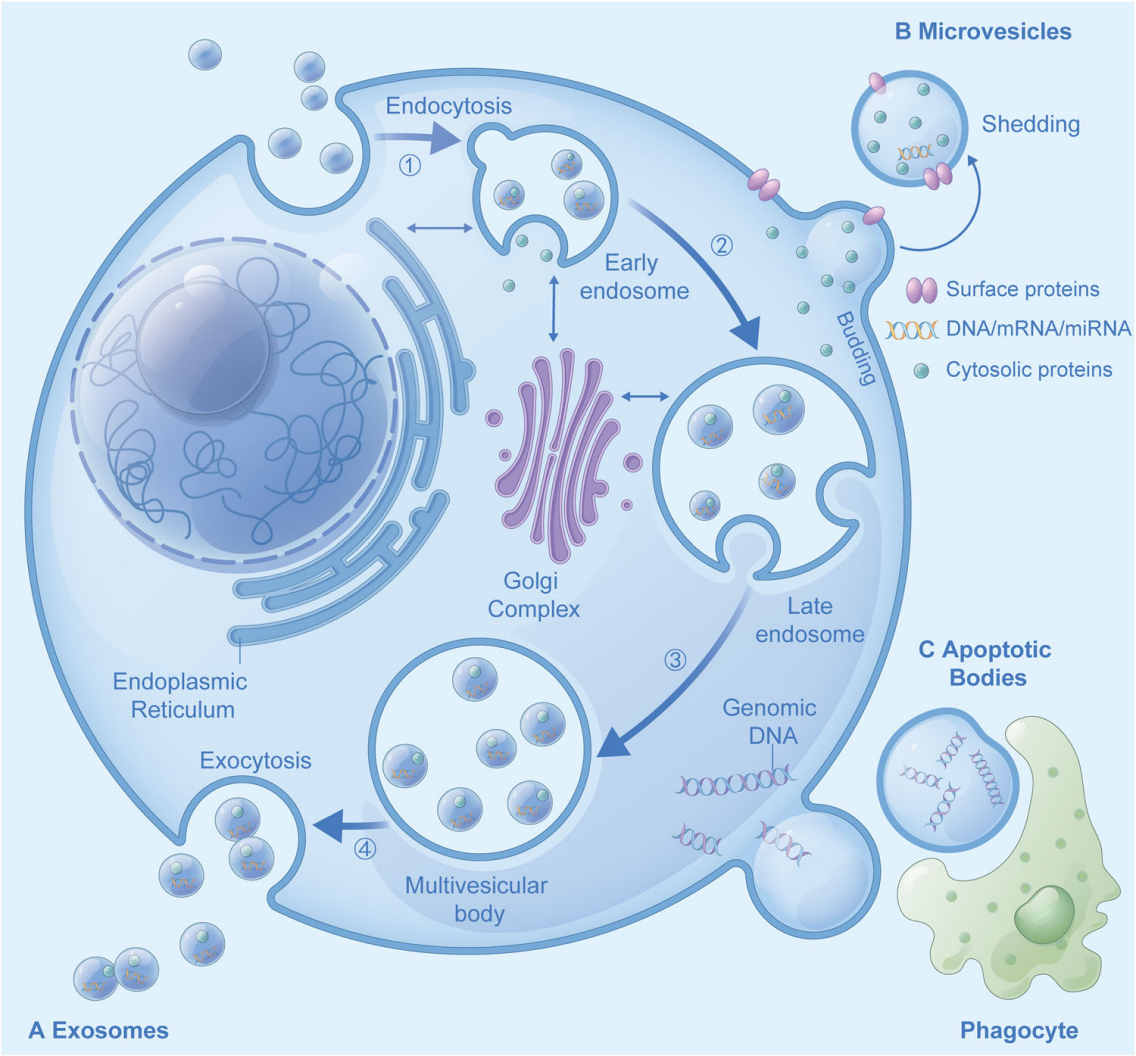
(A) Biogenesis of exosomes: (1) membrane invagination forms early endosomes, (2) early endosomes were loaded with particulate matter, (3) late endosomes and multivesicular body were formed successively, (4) multivesicular body membrane fuses with the cell membrane and releases intracavitary vesicles (exosomes) through exocytosis; (B) microvesicles (EVs): cell membrane sprouts or bubbles to form microvesicles; (C) apoptotic bodies: cells that initiate the apoptotic process form apoptotic bodies by germinating and shedding or forming autophagosomes, which are eventually engulfed by phagocytes.

Exosomes are small membrane vesicles with diameters of 30–150 nm. They are naturally found in body fluids, such as blood, cerebrospinal fluid (CSF), saliva, breast milk, alveolar fluid, bile, joint fluid, amniotic fluid, semen, and urine. They were first discovered in 1983 from sheep's red blood cells before Johnstone coined “exosomes” in 1987.^84^, ^85^ In vitro experiments demonstrated that almost all cultured cells could secrete exosomes under normal and pathological conditions.^86^ As EVs, exosomes are relatively similar to microvesicles and apoptotic bodies (Figure 1).^87^ In contrast, the composition and structure of exosomes are more complex and contain additional components from the extracellular matrix.^88^

**FIGURE 2:**
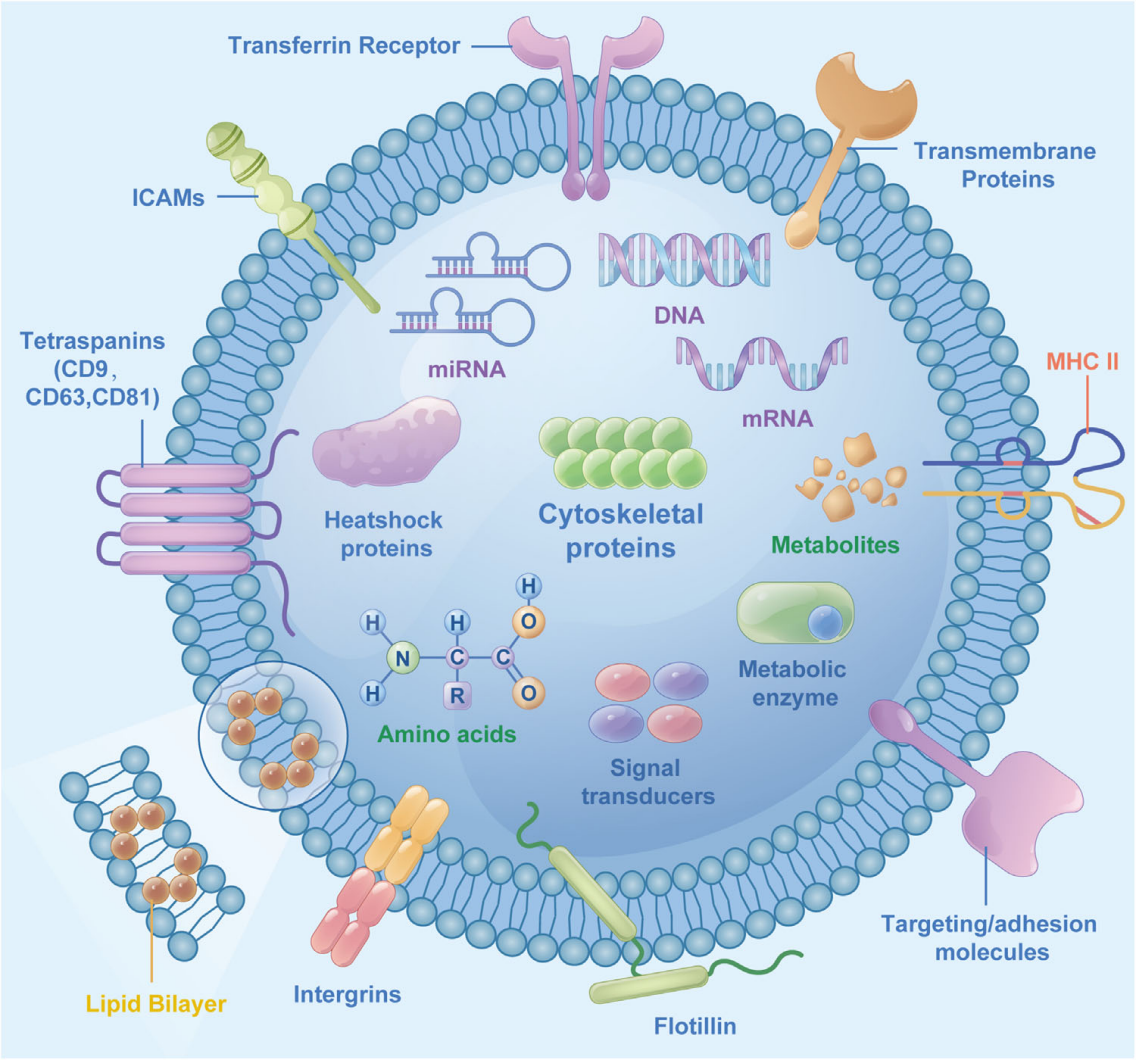
Exosome composition: (1) Lipids: lipid bilayers form the exosome membrane (yellow label); (2) nucleic acids: DNA and RNA (mRNA, miRNA) (purple label); (3) immunoregulatory molecules: major histocompatibility complex (MHC): MHC‐I, MHC‐II (orange label); (4) proteins: transmembrane proteins: tetraspanins (CD9, CD63, CD81), adhesion factors: intercellular adhesion molecules (ICAM), integrin, transferrin receptor, flotillin; heat shock protein, cytoskeletal protein, metabolic enzymes, signal transducers (blue label); (5) metabolites: derived from maternal cells and the surrounding microenvironment, such as amino acids (green label).

Lipids and proteins are the main components of exosomal membranes and are involved in the formation and release of exosomes.^89^, ^90^, ^91^, ^92^ Exosomes contain essential biologically active molecules^93^, ^94^ (Figure 2, Table 1). Lipid bilayers can stabilize the activity of biological molecules such as nucleic acids and proteins contained in these exosomes.

**TABLE 1:** General composition of exosomes.

Composition	Samples	Reference
Lipids	Ceramide, cholesterol, GM3, phosphatidylserine, phosphoglycerides	2,5,10,93
	Phosphatidylcholine, phosphatidylethanolamine, phosphatidylinositol, sphingomyelin	2,60,89
Nucleic acids	RNA: mRNA, ncRNA (microRNA, lncRNA, circRNA)	2,5,50,94
	DNA: tumor susceptibility genes	2,89,94
Immune regulatory molecules	MHC: MHC‐I, MHC‐II	2,49
Proteins	Inner membrane fusion/transport proteins: annexins, Rab proteins	2,10,93
	Cytoskeletal proteins: tubulin protein, actin protein	9,10,93
	Heat shock proteins: Hsp70, Hsp90	9,10
	A variety of metabolic enzymes: peroxidase, GAPDH, DPP4	9
	Involved in the formation of multiple vesicle bodies: Alix, TSG101	2,9
	Integrins and the four transmembrane protein family: CD9, C063, CD81, CD82	2,9
	Signal transduction proteins: protein kinases, G proteins	10,89
Metabolites	Derived from maternal cells and the surrounding microenvironment	10

Abbreviations: circRNA, circular RNA; lncRNA, long noncoding RNA; MHC, major histocompatibility complex; mRNA, messenger RNA; ncRNA, noncoding RNA.

The various RNAs contained in exosomes are essential for the regulatory role of exosomes.^95^, ^96^, ^97^ MicroRNAs (miRNAs or miR) are highly conserved noncoding single‐stranded RNAs (20–22 nucleotides in length) encoded by endogenous genes.^98^ The binding of miRNAs onto the 3′ untranslated region of messenger RNA (mRNAs) causes deadenylation and forms RNA silencing complexes, which results in mRNA degradation and translation inhibition.^99^, ^100^ Long noncoding RNA (lncRNA) is a type of noncoding RNA (ncRNA) with a length of more than 200 nucleotides that induces DNA‐related effects that regulate transcription, epigenetics, protein/RNA stability, translational, and posttranslational modification.^101^ Circular RNA (circRNA) is a covalently closed single‐stranded RNA that acts as a transcriptional regulator, miRNA sponge, and protein scaffold.^102^, ^103^, ^104^ Its unique circular structure makes it less susceptible to degradation by RNA hydrolases.^105^, ^106^, ^107^ Hence, circRNAs have a longer half‐life than linear RNAs, making them potential biomarkers and therapeutic targets.

### 2.2 Mechanism of action of exosomes in the brain

**FIGURE 3:**
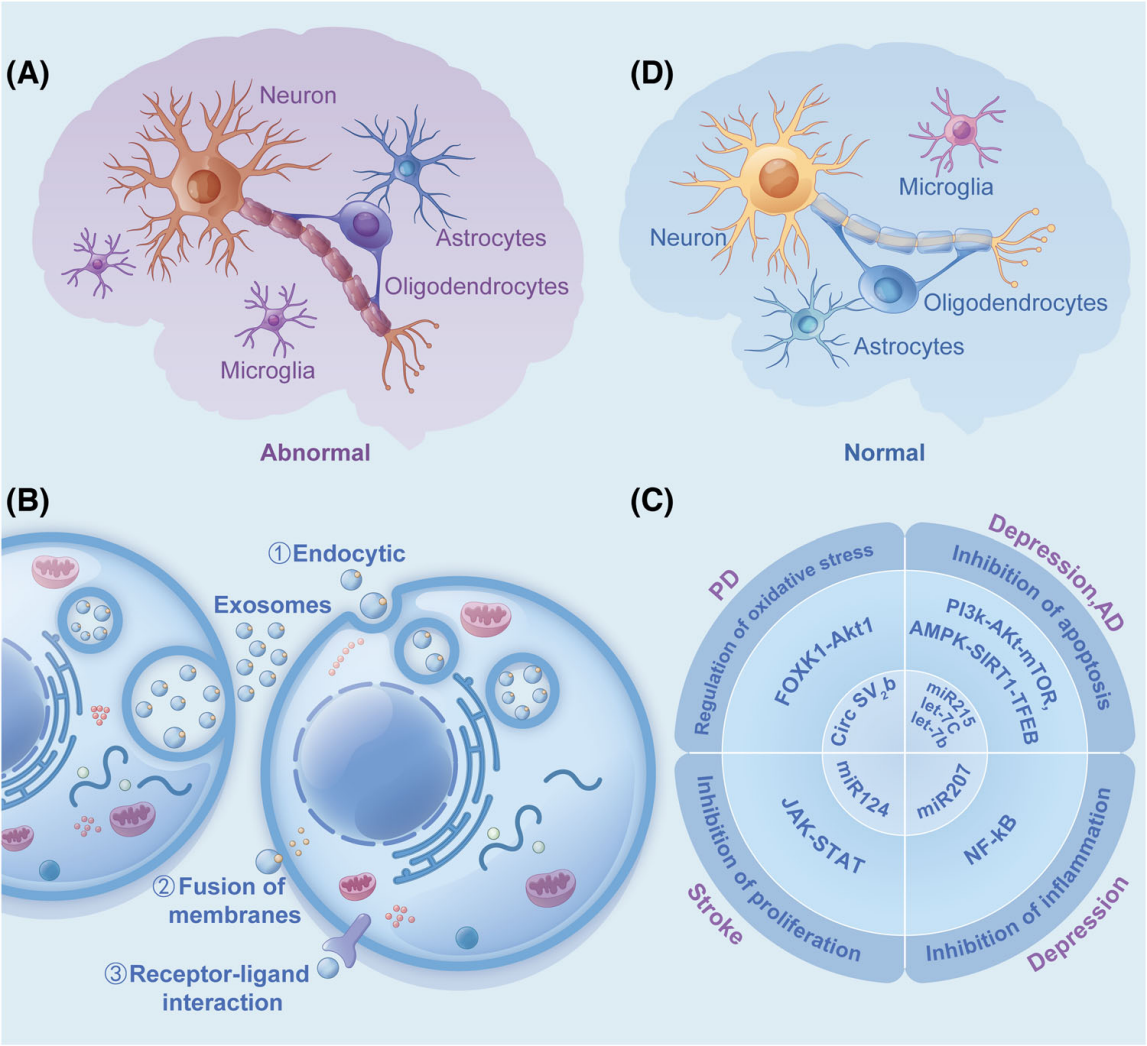
(A) Nerve cells that undergo inflammation and oxidative stress; (B) exosomes interact with target cells; (C) exosomes mediate intracellular signaling pathways; (D) nerve cells that have experienced biological effects.

The structure and function of the brain are complex. Research on the molecular mechanism of encephalopathy is still in its infancy, restricting the development of diagnosis and treatment.^108^ Exosome study in encephalopathy has attracted extensive attention in recent years.^109^, ^110^ Maintaining the normal physiological function of the brain depends on intercellular communication.^111^ Exosomes of various cell types can penetrate the blood–brain barrier and act as important messengers in brain cell–cell communication,^112^, ^113^, ^114^ regulating physiological and pathological processes in the brain.^115^ Exosomes crosstalk brain cells through three main mechanisms: (1) cell internalization: endocytosis by recipient cells; (2) fusion with the cell membrane: direct fusion with the cell membrane through vesicles; and (3) protein–protein or receptor–ligand interaction: adhesion to the cell surface through ligands.^116^, ^117^, ^118^ The contents of the exosomes are then released into the cytoplasm of receptor cells,^119^ mediating intracellular signaling pathways,^120^, ^121^ and influencing biological effects (Figure 3).

Both let‐7c and let‐7b are involved in the expression regulation of insulin‐like growth factor 1 (IGF‐1) gene in the PI3K‐Akt‐mTOR (phosphatidylinositol trihydroxykinase–protein kinase B‐mammalian target of rapamycin) signaling pathway and are significantly downregulated in patients with major depressive disorder (MDD) as compared to healthy subjects. The overexpression of IGF‐1 relays an antidepressant‐like behavior that promotes brain‐derived neurotrophic factor (BDNF) signal transduction.^122^ In vivo experiments demonstrated that exosomes with low levels of miR‐207 had reduced antidepressant activity. miR‐207 was overexpressed in normal mouse exosomes, directly targeted toll‐like receptor 4 (TLR4) to regulate TLR4 interactor with leucine‐rich repeats (Tril), inhibit NF‐κB (κ‐light chain was enhanced in B cells activated by nuclear factor) signaling pathway in astrocytes, and reduced the release of proinflammatory cytokines such as interleukin (IL) (IL‐1β, IL‐6) and tumor necrosis factor (TNF)‐α, alleviate depressive symptoms of mice.^123^ In the M2 microglia, exosomal miR‐124 reduces the formation of glial scar after stroke by inhibiting the expression of signal transducer and activator of transcription 3 (STAT3), promotes poststroke recovery, and alleviates the migration and proliferation of astrocytes.^124^, ^125^ In Parkinson's disease (PD) model, circSV2b overexpression can reduce oxidative stress injury, protect dopaminergic neuron loss, maintain nigrostriatal function, and improve motor defects through miR‐5107‐5p‐FOXK1 (fork‐head box protein K1)‐AKT1 (serine/threonine protein kinase) signaling pathways.^126^ miR‐215 targets the apoptosis‐related genes (BCL2L11 and SIRT1) and functions to regulate the AMPK‐SIRT1‐TFEB (adenosine 5′‐monophosphate [AMP] activated protein kinase [AMPK]‐silent message regulatory factor 1‐transcription factor EB) signaling pathway and prevent neuronal apoptosis. The exercise‐induced upregulation of exosomal miR‐215 may have preventive effects on Alzheimer's disease (AD).^127^

However, exosomes can be hostile to or supportive of the brain, depending on the biological signals they carry.^127^, ^128^ Studies have found that exosomes are involved in the occurrence and development of various encephalopathies,^129^, ^130^, ^131^, ^132^, ^133^, ^134^ providing biomarkers and therapeutic targets for disease diagnosis and treatment.^135^, ^136^, ^137^ Analysis of exosomes in body fluids can identify the subtle changes in the pathophysiological process of neuropsychiatric disorders, providing new means for diagnosing and predicting diseases and a new perspective for treatment.

## 3 EXOSOMES IN NEUROPSYCHIATRIC DISORDERS

**FIGURE 4:**
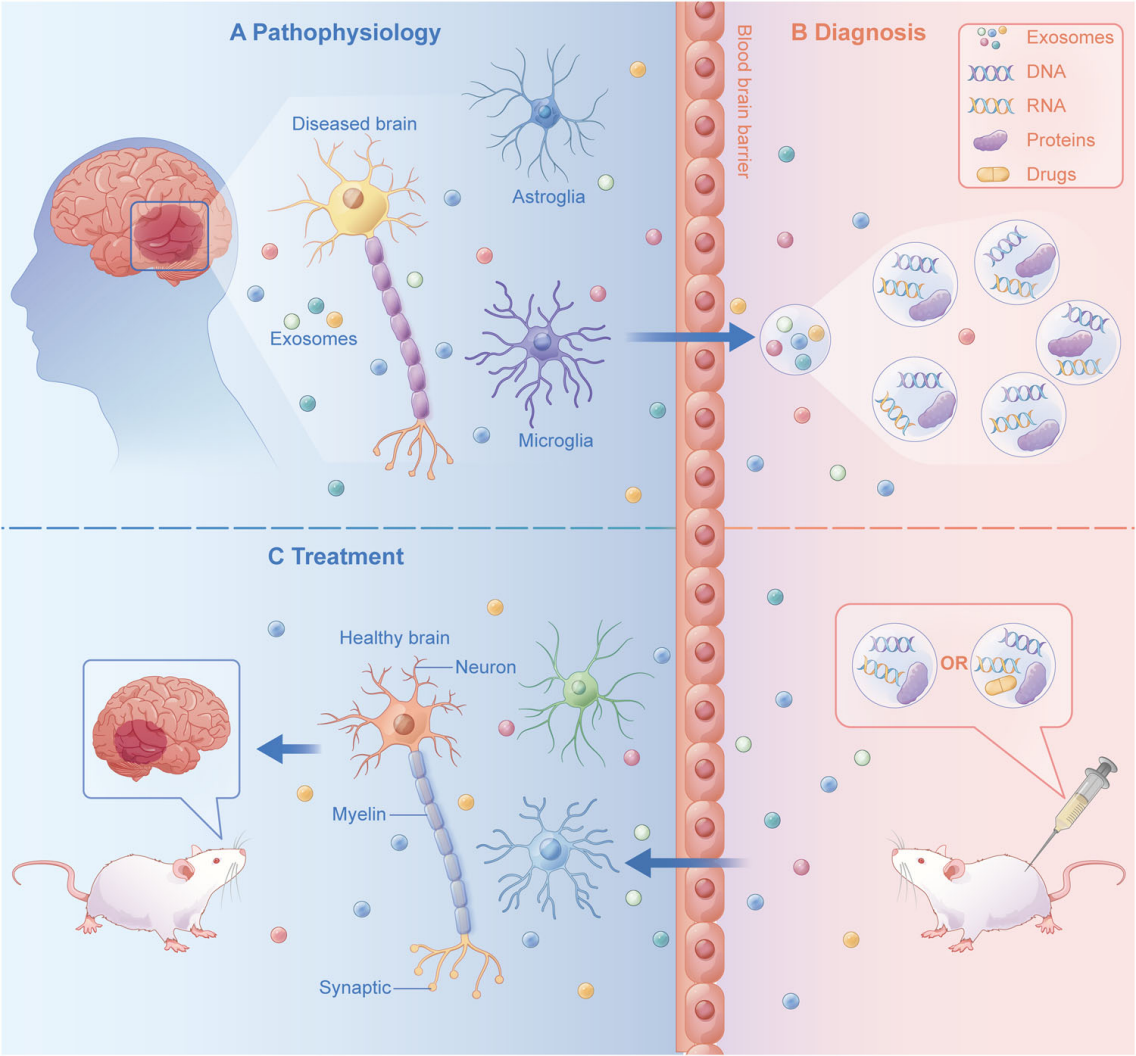
(A) Exosome crossing: exosomes from diseased nerve cells cross the blood–brain barrier and enter the peripheral circulation, releasing their contents such as RNA and protein; (B) extraction and modification of exosomes: exosomes are extracted from body fluids. Their contents are analyzed by high‐throughput sequencing qRT‐PCR and quantitative analysis tools to find clinical biomarkers, diagnose and predict the occurrence and development of diseases, provide a basis for clinical decision‐making, and identify and develop treatments based on exosome contents, such as increasing or decreasing a specific substance, or introduce therapeutic drugs, modify exosomes into engineered exosomes, and inject the engineered exosomes into model animals; (C) therapeutic effects of exosomes: engineered exosomes carrying therapeutic substances enter model animals, cross the blood–brain barrier, and reach the center to play a therapeutic role and restore nerve cell health.

Brain cells damaged by inflammation and oxidative stress exhibit morphological abnormalities and dysfunction. Exosomes derived from diseased nerve cells cross the blood–brain barrier into the peripheral circulation to release their contents, which can be used as biomarkers to diagnose and predict diseases. At the same time, exosomes with altered contents are modified, or therapeutic drugs are loaded into exosomes. As a natural source of body fluids, exosomes cross the blood–brain barrier and participate in the pathophysiological processes of neuropsychiatric diseases (Figure 4). In summary, altered exosomes in disease states can be used as potential biomarkers for disease diagnosis (Table 2). Engineered exosomes can also be used as carriers of bioactive molecules involved in signaling pathways and become potential targets for disease treatment (Table 3). This section summarizes the progress of exosomes' role in diagnosing and treating various neuropsychiatric disorders.

**TABLE 2:** Exosomal contents with pathogenicity in neuropsychiatric disorders.

Disease	Exosome derivation	Contents	Pathogenesis	References
Depression	Serum from patients with depression	miR‐9‐5p	Promote M1 polarization of microglia cells	144
Schizophrenia	Plasma from EPP	miR‐137	Induce autophagy and aggravating nerve damage	152
	Plasma from patients with TRS	miR‐675‐3p	Involved in neuroapoptosis	154
PTSD	Peripheral blood of soldiers with mTBI	NFL	Involve in neurodegenerative and inflammatory processes	168,169
AD	Plasma of Chinese AD patients	miR‐185‐5p	Involve in the production and accumulation of Aβ	186,187
PD	Serum from PD patients	TNF‐α, IL‐1β	Induce dopaminergic neuron death	166
ALS	Motor neurons of fALS	miR‐625‐3p	Mediate neuroinflammation, oxidative stress and apoptosis	243
Epilepsy	Plasma from patients with mTLE + HS	hsa‐miR‐184	Modulate immunity, inflammation and apoptosis, and exacerbates neuronal death	261
Stroke	Serum from AIS patients	miR‐9	Involved in the inflammatory process, regulating angiogenesis	268
	Serum from AIS patients	circ_0006896	Promote vascular migration and proliferation, resulting in plaque instability	273

Abbreviations: AD, Alzheimer's disease; AIS, acute ischemic strokes; ALS, amyotrophic lateral sclerosis; Aβ, β‐amyloid; EPP, early psychosis patients; fALS, familial ALS; hsa: *Homo sapiens*; IL, interleukin; mTBI, mild traumatic brain injury; mTLE + HS, mesial temporal lobe epilepsy with hippocampal sclerosis; NFL, neurofilament; PD, Parkinson's disease; PTSD, posttraumatic stress disorder; TNF, tumor necrosis factor; TRS, treatment‐resistant schizophrenia.

**TABLE 3:** Exosomal contents with therapeutic potential in neuropsychiatric disorders.

Diseases	Exosome derivation	Contents	Therapeutic targets	References
Depression	Patients with refractory depression	let‐7c and let‐7b	Inhibit apoptosis	122
	Non‐stressed mouse NK cells	miR‐207	Inhibit inflammation	123
	BMSCs	miR‐26a	Improve hippocampal neuron damage	147
AD	Blood	miR‐215	Prevent neuronal apoptosis	188
	BMSCs	miR‐21, miR‐29b, and miR‐146a	Improve synaptic dysfunction and suppress inflammation	191
	ADSCs	miR‐770‐3p	Promote the transformation of hippocampal microglia from M1 to M2 phenotype	193
	M2 microglia	miR‐124‐3p	Inhibit neuronal inflammation and promote nerve growth	194
PD	ADSCs	miR‐188‐3p	Reverse the apoptosis	205
	Serum of PD mice	CircSV2b	Resist oxidative stress injury	206
HD	ADSCs	Neurotrophic factor	Reduce mHTT aggregation, mitochondrial dysfunction and apoptosis	223,224
	Astrocytes	αB‐Crystallin	Reduce the aggregation of mHTT and the cytotoxicity of misfolded proteins	225
ALS	Mouse ADSCs	SOD‐1	Regulate SOD aggregation, participate in cell adhesion	252
Stroke	Astrocytes	miR‐190b	Regulate autophagy	278
	Microglial	miR‐21	Inhibit inflammation	280
	BMSCs	miR138‐5p	Promote astrocyte proliferation, inhibit inflammation and apoptosis	281
	BMSCs	miR‐133b	Promote functional recovery	282
	BMSCs	miR‐21‐5p	Promote neurological function	282
	BMSCs	miR‐150‐5p	Inhibit TLR5, protect cerebral I/R injury	283
	M2 microglial	miR124	Inhibit proliferation	125
	BMSCs	miR‐124‐3p	Reduce neuronal damage, inhibit oxidative stress, and reduce neuronal apoptosis	284,285
	NSCs	miR‐150‐3p	Reduce infarct size and inhibit neuronal apoptosis	286

Abbreviations: AD, Alzheimer's disease; ADSCs, adipose‐derived stem cells; ALS, amyotrophic lateral sclerosis; BMSCs, bone mesenchymal stem cells; circ, circular; HD, Huntington's disease; I/R, ischemia/reperfusion; mHTT, mutant huntingtin; NK cells, natural killer cells; NSCs, neural stem cells; SOD, superoxide dismutase; TLR, toll‐like receptor.

### 3.1 Depression

Mental disorders constitute a global health burden, with depression among the common one.^138^ Depression is the second leading cause of disability in China and is normally characterized by low mood, loss of interest, fatigue, and in some cases, suicidal tendencies.^139^ The currently available treatments for depression include medication, psychotherapy, and physical therapy. Nevertheless, the cause of depression remains unclear, with limited treatment efficacy and a high recurrence rate.

Blood exosomes from depression patients induced depression‐like behavior in normal mice.^140^ Compared with healthy controls, plasma exosomes of patients with refractory depression were significantly upregulated by *Homo sapiens* (hsa)‐miR‐335‐5p and downregulated by hsa‐miR‐1292‐3p. These two miRNAs are associated with synaptic functions and treatment‐resistant depression.^141^ The expression levels of miRNAs (hsa‐miR‐16‐5p, hsa‐miR‐129‐5p, hsa‐miR‐363‐3p, and hsa‐miR‐92a‐3p) in exosomes from drug‐dependent depressed patients were negatively correlated with both the Hamilton Rating Scale for Depression (HRSD) and Hamilton Anxiety Rating Scale (HARS).^142^ Some studies isolated serum exosomes from patients with severe depression and healthy controls, and the expression level of miR‐146a was higher in patients with severe depression than in healthy controls. Compared with the non‐remission group, the levels of let‐7e, miR‐21‐5p, miR‐145, miR‐146a, and miR‐155 were significantly decreased before treatment in the remission group and increased after antidepressant treatment.^143^ These exosomal miRNAs may play an important role in predicting the effects of antidepressants. There were significantly higher miR‐9‐5p expression levels in serum exosomes from depressed patients. miR‐9‐5p was transferred from neurons to microglia through exosomes, leading to microglia M1 polarization, further neuronal injury, and exacerbated depressive symptoms.^144^ Genome‐wide miRNA expression profiling of blood‐derived exosomes from depressed patients and healthy subjects reported that the most differentially expressed exosomal miRNA, hsa‐miR‐139‐5p, was upregulated and could distinguish patients with MDD from controls. Exosomes from the blood of patients with depression can induce depression‐like behavior in normal mice by tail vein injection. However, the injection of blood exosomes isolated from healthy volunteers into chronic unpredictable mild stress (CUMS) mice alleviated depression‐like behavior. CUMS mice also exhibited significantly higher levels of exosomal miR‐139‐5p in the blood and brain. Treatment with exosomes from healthy blood and miR‐139‐5p antagonists both increased hippocampal neurogenesis in CUMS mice, whereas treatment with exosomes from depressed patients decreased hippocampal neurogenesis in normal mice.^145^, ^146^ Therefore, exosomal miRNAs may be potential targets for the diagnosis and treatment of depression.

Bone marrow stem cell (BMSC)‐derived exosomes (BMSC‐Exos) improved hippocampal neuronal injury in depression‐induced rats by upregulating miR‐26a.^147^ miR‐207 is overexpressed in normal mouse exosomes, inhibiting the NF‐κB signaling pathway in astrocytes, reducing the release of pro‐inflammatory factors, and alleviating depressive symptoms in mice.^123^ Plasma exosomes exerted antidepressant‐like effects on sigma‐1 receptors in lipopolysaccharide (LPS)‐induced depression.^148^, ^149^ Therefore, further research can facilitate the development of new exosome‐based treatments for MDD in the future.

### 3.2 Schizophrenia

Schizophrenia (SCZ) is a chronic mental illness of unknown etiology, affecting nearly 1% of the population. It mostly affects young adults and the elderly, with symptoms such as irregularities in physical behavior, logical thinking, and emotional experience. Antipsychotics have limited effects on their treatment and severe psychiatric and metabolic side effects.^150^, ^151^ Likewise, these drugs mainly improve positive symptoms such as hallucinations and thought disorders, but not negative (emotional flatness and social withdrawal) and cognitive (learning and attention disorders) symptoms.

The identification of biomarkers in the early stages of SCZ can help elucidate its pathogenesis and treatment. The expression of exosomal miR‐137 increased, whereas COX6A2 decreased in patients with early psychosis. This induces autophagy by regulating the expression of mitophagy markers (Fundc1, NIX, and LC3B), and aggravates the damage of parvalbumin interneurons in the prefrontal cortex of mice under oxidative stress.^152^ Hence, the level of plasma exosomal miR‐137/COX6A2 is closely related to the pathology of SCZ and can be used as a biomarker for early diagnosis. BDNF plays a vital role in the development and maintenance of the nervous system. The miRNA expression profile of serum exosomes in patients with SCZ reported that hsa‐miR‐206 directly targets the BDNF mRNA, which negatively affects cognitive functions.^153^ Microarray analysis indicated that 13 miRNAs were upregulated, and 18 miRNAs were downregulated in plasma exosomes of treatment‐resistant SCZ (TRS) patients. The target genes (PTRO, SRSF2, and CPEB4) were associated with neuronal apoptosis and brain development. The expression of miR‐675‐3p was upregulated in TRS patients and exosomes of clozapine‐treated SH‐SY5Y cells. The results suggested that clozapine can affect the expression of exosomal miRNAs.^154^ miR‐223 is enriched in astrocytes and highly expressed in glial and neuronal exosomes. The levels of miR‐223 in the orbitofrontal cortex were significantly increased after SCZ patient deaths. miR‐223 is secreted by exosomes and enriched in astrocytes, which targets glutamate ionotropic receptor NMDA (*N*‐methyl‐d‐aspartic acid) type subunit 2B (GRIN2B) and glutamate ionotropic receptor AMPA (alpha‐amino‐3‐hydroxy‐5‐methyl‐4‐isooxazopropionic acid) type subunit 2 (GRIA2), its expression was significantly increased in SCZ patients. Studies have shown that miR‐223 inhibited the expression of neuronal glutamate receptor genes through specific binding to mRNA sites, thereby reducing the levels of GRIN2B and GRIA2 in mouse hippocampal neurons and decreasing synaptic activity.^155^ This may be a mechanism of the pathophysiology of SCZ. Low NMDA receptor signaling is consistent with cognitive and behavioral disorders in SCZ.^156^

Compared with healthy controls, plasma exosomes of SCZ patients showed significantly higher concentrations of glial fibrillary acid protein (GFAP) and lower concentrations of α‐II‐spectrin. Upregulation of GFAP indicates astrocyte injury. α‐II‐Spectrin is a cytoskeletal protein that makes up axons and synapses. It is abundant in brain neurons, and its downregulation suggests sustained nerve cell damage.^75^ Therefore, an in‐depth study of protein and miRNA contents in exosomes can better elucidate the pathogenesis of SCZ and provide a basis for better treatment strategies.

### 3.3 Autism spectrum disorder

Autism spectrum disorder (ASD) is a central neurodevelopmental disorder caused by various genetic and environmental factors. ASD usually develops within 3 years of age, and its main symptoms include social communication disorder, communication disorder, and cognitive inflexibility.^157^ ASD lacks specific treatments and requires lifelong care and maintenance.

Bioinformatics has revealed the genes and biological networks associated with ASD.^158^ An exosomal proteomic analysis in an animal model of Rett syndrome–ASD suggested that the disruption of MECP2 function can affect brain development.^159^ Active synaptic vesicle (SV)‐associated transcripts (SVATs) are present in the central nervous system (CNS) and the peripheral circulation, which play an important role in SV release and recycling. Abnormal SVAT expression has been found in patients with ASD. Alterations in the genetic expressions of lncRNAs and mRNAs related to SVATs were detected in the peripheral blood exosomes of children with ASD.^160^ Thus, monitoring SVAT changes during pregnancy can predict the development and function of neural circuits in the fetal brain.

Elevated levels of proinflammatory cytokines and abnormal microglia/astrocyte responses were observed in the brains of animal models of ASD, suggesting that inflammation is involved in the pathogenesis of ASD.^161^ The pathogenesis of ASD is associated with dysfunctional inflammatory pathways and macrophages. Studies have validated that the increased level of IL‐1 in exosomes of ASD patients regulates inflammation via miRNAs.

Stem cell‐derived exosomes polarize macrophages toward the M2 phenotype and stimulate anti‐inflammatory cytokines/proteins, thus reducing the pro‐inflammatory conditions associated with ASD.^162^ Exosomal‐content recipient cells (RNA and protein) have the potential to repair damaged cells in ASD. BTBR T+ tf/J (BTBR) mice exhibit symptoms of ASD, which facilitates the assessment of ASD behaviors. In a BTBR mouse model, exosomes derived from mesenchymal stem cells (MSCs) that cross the blood–brain barrier and enter the brain via nasal administration ameliorated ASD‐related behaviors and symptoms.^163^, ^164^ Transplantation of human BMSCs (bone MSCs) into the lateral ventricles of BTBR mice resulted in the long‐term improvement of the behavioral phenotype of autism, and exosomes were the primary mediators of the therapeutic effect.^157^ The beneficial effect of exosomes in BTBR mice may be a novel noninvasive treatment strategy for ASD symptoms.

### 3.4 Posttraumatic stress disorder

Posttraumatic stress disorder (PTSD) refers to a mental disorder related to trauma and stress that develops after experiencing a traumatic event.^165^, ^166^ The common symptoms are traumatic reexperience, avoidance, negative cognitive and emotional changes, and increased alertness.

PTSD is associated with mild traumatic brain injury (mTBI). A specific type of neurofilament (NFL) miRNA is associated with neurodegenerative and inflammatory processes and is a major component of the neuronal cytoskeleton. This miRNA is particularly expressed in myelinated nerve axons of large neurons, and its elevated expression level is a hallmark of axonal injury.^167^ The levels of hsa‐miRNA‐139‐5p, tau, Aβ42, IL‐10, and NFL were elevated in peripheral blood EVs of soldiers with mTBI. As a significant component of the neuronal skeleton, NFL is a marker of axonal damage. It is involved in neurodegenerative and inflammatory processes and correlates with the severity of PTSD symptoms.^168^, ^169^ NFL in exosomes may be a new diagnostic tool and therapeutic target for PTSD.

### 3.5 Alzheimer's disease

AD is a progressive neurodegenerative disease with insidious onset and is characterized by memory, cognitive and behavioral impairments. Dementia affected approximately 50 million people in 2018, which is projected to triple by 2050.^170^ Its pathogenesis is associated with a variety of factors, including genetic alterations, deposition of β‐amyloid (Aβ) protein, accumulation of tau into neurofibrillary tangles in neurons, and abnormal phosphorylation of tau. The accumulation of Aβ 1–42 in the brain and the phosphorylation of tau protein are the two main pathological features of AD.^171^

Exosomes are involved in neuroinflammation,^172^ productions and clearance of Aβ,^173^, ^174^ transport of Aβ, tau, prions, and α‐synaptic nucleoproteins,^175^, ^176^ which trigger the hyperphosphorylation of Aβ and tau.^177^ Intracranial injection of exosomes from AD mouse brains in wild‐type (WT) mice resulted in mitochondrial damage and neurotoxicity. The levels of tau protein and Aβ1–42 in exosomes of the AD group were significantly increased.^115^ Exosomal tau protein can be used as biomarkers for the preclinical diagnosis of AD.^178^ Aβ1–42, t‐tau, p‐T181‐tau, and P‐S96‐tau may be used as biomarkers for AD.^179^

More and more evidences emphasize the regulatory role of miRNAs in the pathogenesis of AD. The expressions of miR‐135a and miR‐384 were upregulated, whereas miR‐193b was downregulated in the serum exosomes of AD patients.^180^ Likewise, miR‐342‐3p was downregulated in plasma exosomes, whereas miR‐212 and miR‐132 were downregulated in neural exosomes.^181^ miRNAs, such as miR‐9‐5p, miR‐598, miR‐125b, miR‐29, miR‐342‐3p, and miR‐193b, are highly stable and resistant to degradation in exosomes before the onset of AD, suggesting their promising roles as biomarkers for the early clinical diagnosis and monitoring of AD.^182^, ^183^ miR‐29c‐3p, miR‐384, and nerve cell adhesion molecule in double‐labeled exosomes have the potential for early diagnosis of AD.^184^, ^185^ Serum exosomal miR‐185‐5p was significantly downregulated in AD patients and mice as compared with the control group. Overexpression of neuronal APP (amyloid precursor protein) regulates the levels of miR‐185‐5p in exosomes, thereby preventing the miR‐185‐5p‐mediated translational repression of APP transcripts. The regulatory role of exosomes in APP expression suggests that exosomes and their miRNAs may be potential therapeutic targets and biomarkers for the treatment and diagnosis of AD.^186^, ^187^ The exercise‐induced upregulation of exosomal miR‐215 had preventive effects on AD.^188^ Compared with normal controls, the expression of circular AXL (circAXL) in the exosomes of AD serum samples was significantly increased, whereas the expression of miR‐1306‐5p was significantly decreased, indicating the prospective diagnosis of AD. An AD cell model was constructed by treating SK‐N‐SH cells with Aβ1–42, and the results indicated that circAXL and PDE4A (phosphodiesterase 4A), a key regulator of cAMP (cyclic adenosine monophosphate) degradation, was upregulated, whereas the expression of miR‐1306‐5p was downregulated. CircAXL participates in Aβ1–42‐induced neuronal injury by targeting the miR‐1306‐5p/PDE4A axis. CircAXL knockdown can inhibit PDE4A by releasing miR‐1306‐5p, reduce apoptosis, inflammation, oxidative stress, and endoplasmic reticulum stress in AD cells, and reduce Aβ1–42‐induced neurotoxicity in AD pathology.^189^ This approach provides a new treatment concept for AD.

The proteins contained in exosomes can be used as biomarkers of AD. The SNAP25 (synaptosome‐associated protein of 25 kDa) and synaptophysin‐I binding protein in neuron‐derived exosomes can predict the development of AD. The levels of complement proteins (C1q, C3b, and C3d) and cytokines (IL‐6, IL‐1β, and TNF‐α) in astrocyte‐derived exosomes (ADEs) were significantly different between AD patients and healthy controls.^181^ AD patients had higher levels of complement proteins in ADEs when compared with healthy controls. However, the ADEs of complement regulatory proteins (CD59, CD46, decay‐accelerating factor, and complement receptor type 1 [CR1]) were lower in AD patients than in healthy controls and further decreased as the disease progressed. Thus, measuring complement protein levels in exosomes can predict disease progression. Growth‐associated protein‐43 (GAP‐43), neurogranin, synaptotagmin (Rab3A), and SNAP25 in neurogenic exosomes have been studied as blood biomarkers for AD and mild cognitive impairment.^134^

Different exosomes injected into AD mouse models exert different therapeutic effects.^81^, ^190^ BMSC‐Exos contain miR‐21, miR‐29b, and miR‐146a, which can improve synaptic function and inhibit inflammation, thus improving cognitive function in AD animal models.^191^ BMSC‐Exos isolated from the femur and tibia of adult C57BL/6 mice can improve the AD‐like behavior of the streptozotocin‐injected mouse model. The mechanism is related to the inhibition of microglial activation and astrocytes in the hippocampus of the mouse model. The expressions of neuroinflammatory factors (IL‐1β, IL‐6, TNF‐α, Aβ1–42, and p‐tau) decreased, while the protein expressions of synaptophysin and BDNF were upregulated.^192^ Hypoxia‐treated adipose‐derived stem cell (ADSC)‐derived exosomes (ADSC‐Exos) promoted the transformation of hippocampal microglia from the pro‐inflammatory M1 phenotype to the anti‐inflammatory M2 phenotype in AD mice, targeted miR‐770‐3p and TREM2 (trigger receptor 2 expressed in bone marrow cells), reduced damage to hippocampal neurons, and improved cognitive function.^193^ On the contrary, the administration of M2 microglia‐derived exosomes (M2‐Exos) in AD cell models (HT‐22 cells and MAP2‐positive neuronal cells of APP/PS1 AD mice models) reduced Aβ plaque deposition and oligomer expression, partially improved cell viability, and restored the mitochondrial membrane potential. In addition, it can reduce the accumulation of reactive oxygen species (ROS) in mitochondria and cells in a dose‐dependent manner and play a neuroprotective role. Exosomal miR‐124‐3p can inhibit neuronal inflammation and promote neuronal growth. Similarly, miR‐124 can change the level of autophagy in APP/PS1 mice and alleviate the pathological progression of AD. Therefore, we hypothesize that the protective effect of M2‐Exos in AD may be mediated by miR‐124.^194^ Exosomes from human amniotic fluid stem cells (hAFSC‐Exos) targeting LPS‐activated BV‐2 microglia as a model of neuroinflammation. A study was conducted to investigate the effect of exosomes on the SH‐SY5Y interaction between AD neurons and microglia. The results demonstrated that hAFSC‐Exos could inhibit the inflammatory response, oxidative stress, and microglial apoptosis and could be a potential therapeutic drug for diseases related to inflammation such as AD in the future.^195^

Neuroinflammation is associated with neuronal cell death in AD. Coenzyme Q10 (CoQ10), as a supplement to anti‐inflammatory and antioxidant stress, can improve AD‐related inflammation and oxidative stress. Furthermore, exosomal CoQ10 promotes neuronal differentiation by increasing hippocampal BDNF and SOX2 levels and also enhances AD cognitive and memory deficits.^196^ Quercetin‐loaded exosomes potently ameliorate cognitive function in AD mice by inhibiting Tau phosphorylation and reducing neurofibrillary tangle formation.^197^ With the deepening of the research on engineering exosomes, the optimal ratio of exosome contents may be a new idea for treating AD in the future.

### 3.6 Parkinson's disease

PD, also known as parkinsonism, is a neurodegenerative disease in which a lesion of dopaminergic neurons in the substantia nigra of the midbrain causes a decrease in dopamine levels and prevents the brain from stimulating the muscles.^198^ The neuropathological features of PD commonly include the degeneration of substantia nigra dopaminergic neurons, extensive misfolding and accumulation of α‐synuclein (α‐syn), and the formation of eosinophilic inclusion bodies (Lewy bodies) in the cytoplasm of residual substantia nigra neurons.^199^ Genetic mutation, α‐syn, oxidative stress, mitochondrial dysfunction, autophagy, and infection are hallmarks in the pathogenesis of PD.

Glucocerebrosidase (GBA) gene mutation is the most common genetic risk factor for PD. GBA is cleaved into glucose and ceramide, and ceramide is involved in exosome biogenesis. GBA activity decreases in PD patients, resulting in significant increases in the number of exosomes released from the brain.^200^ POU3F3 in neurogenic exosomes, containing L1 cell adhesion molecule, is significantly positively correlated with α‐syn and negatively correlated with the activity of lysosomal enzyme β‐GBA encoded by the gene, which can lead to the degeneration of dopamine neurons and accelerate the progression of PD.^200^, ^201^

Exosomes are involved in the internalization and transport of α‐syn oligomer. α‐syn aggregation is a result of exosome internalization, which leads to the loss of dopaminergic neurons and motor dysfunction. α‐syn oligomers can be transferred in exosomes from damaged neurons to normal neurons.^181^ This promotes the transmission of pathological synuclein and induces aggregation and neuronal apoptosis, thus promoting the pathological changes of PD.^202^ α‐syn oligomers are present in microglia/macrophage‐derived exosomes in the CSF of PD patients, where they aggregate and multiply through dysregulation of autophagy. A study injected exosomes containing α‐syn from PD patients into the striatum of mice and reported dopaminergic neuronal degeneration and motor deficits.^203^ The ratio of α‐syn oligomers to α‐syn in plasma exosomes was correlated with the severity of PD.^204^

Abnormal miRNA expression levels in serum and CSF‐derived exosomes in PD patients can be used as biomarkers for PD diagnosis. In PD patients, the expression levels of exosomal let7f‐5p and miR‐125a‐5p were increased, whereas the expression levels of miR‐27a‐3p, miR‐423‐5p, miR‐151a‐3p, miR‐1, and miR‐19b‐3p were decreased.^205^ The reduced expression of miR‐23b‐3p in PD exosomes led to the upregulation of α‐syn.^206^ miR‐151a‐5p, miR‐24, miR‐485‐5p, miR‐331‐5p, miR‐214, miR‐99a‐5p, miR‐126‐5p, miR‐1, miR‐19b‐3p, miR‐153, miR‐409‐3p, miR‐10a‐5p, and miR‐501‐3p were upregulated in the serum and CSF‐derived exosomes of PD patients and could be used as biomarkers for the diagnosis of PD.^207^ LncRNAs are enriched in dopaminergic neurons, which can also be used as diagnostic markers of PD. When compared with healthy controls, one mRNA (NDUFB7) and three lncRNAs (ENST00000564683, ENST00000570408, and ENST 00000628340) extracted from blood exosomes of PD patients were differentially expressed. NDUFB7, which encodes reduced nicotinamide adenine dinucleotide ubiquitin oxidoreductase (Complex I), is positively correlated with the three lncRNAs. These are markers related to mitochondrial respiration and may be a potential biomarker to evaluate the effect of mitochondrial function recovery in PD rehabilitation.^208^ These findings provided valuable potential biomarkers for evaluating future treatment strategies for PD. Overexpression of TNF‐α and IL‐1β in serum exosomes of PD patients directly induced dopaminergic neuron death.^166^ Glial exosomes containing inflammatory molecules can communicate with neurons and promote the development of PD.

Regarding treatment, exosomes can be used as drug carriers for PD, with a natural brain‐targeting ability. Dopamine‐loaded exosomes reported better therapeutic efficacy and lower systemic toxicity than direct intravenous dopamine administration in the PD mouse model. In PD models, exosomes are delivered with therapeutic catalase mRNA to attenuate neurotoxicity and neuroinflammation.^209^, ^210^, ^211^ Blood‐derived exosomes from healthy volunteers can reduce dopaminergic neuron damage and improve the motor coordination ability of PD mice. Intracerebroventricular injection of exosomes loaded with an antisense oligonucleotide (ASO)‐4 significantly increased α‐syn aggregation and attenuated dopaminergic neuron degeneration in PD mice, thus improving motor functions.^212^, ^213^ ADSC‐Exos are rich in miR‐188‐3p and can reverse PD‐related apoptosis in animal models.^205^ In the PD model, circSV2b overexpression can reduce oxidative stress injury, protect dopaminergic neuron loss, maintain nigrostriatal function, and improve motor defects through miR‐5107‐5p‐FOXK1‐AKT1 signaling pathways. CircSV2b may help to better understand the pathogenesis of PD and become the new target for the diagnosis and treatment of PD.^126^ These findings provide valuable potential biomarkers for evaluating treatment strategies for PD.

### 3.7 Huntington's disease

Huntington's disease (HD) is a rare monogenic dominant neurodegenerative disease. The cytosine–adenine–guanine (CAG) repeat expansion in exon 1 of the huntingtin gene causes fragmentation and aggregation of mutant huntingtin (mHTT) proteins. This affects neuronal cell function and causes cell death, resulting in cognitive impairment and involuntary movement. HD usually develops in middle childhood with insidious onset, and the main clinical manifestations are involuntary dance movements, cognitive disorders, and neuropsychiatric symptoms.^214^, ^215^, ^216^ In HD, the incomplete expansion of the CAG repeat in exon 1 of the HTT gene results in the production of polyglutamine (polyQ) protein. Both polyQ protein and amplified repeat RNAs can cause toxicity and neurodegeneration.^217^

Huntington protein is a large 350 kDa protein, which makes it difficult to load into exosomes. Unconventional mHTTS contain many duplicates of glutamine, which can be transported, transported, and eventually cause nerve cell death through exosomes.^129^ Exosomes can load, carry, and transfer mHTTS between cells, leading to nerve cell death and causing HD‐related behaviors and pathological manifestations.^218^ Fibroblasts from HD patients have 72, 143, and 180 CAG repeats, whereas induced pluripotent stem cells have 143 CAG repeats. When their exosomes are injected into the ventricles of neonatal WT mice, mHTT can be delivered to genetically unrelated healthy tissues, causing loss of striatal spines. Inflammation in related brain regions increased gliosis, motor and cognitive impairments, HD‐related behavior, and phenotypic characteristics.^219^ The results indicated that exosomes could load mHTT between cells and transmit prion‐like proteins.

Exosomes have recently been identified as carriers of siRNAs (small interfering RNAs). miR‐214, miR‐150, miR‐146a, and miR‐125b in exosomes regulate the HTT gene.^220^ The differential expression of miRNAs in exosomes provides the potential for diagnosing and treating neurodegenerative diseases. Hydrophobic‐modified siRNAs can be efficiently loaded into exosomes during co‐incubation or internalized by mouse primary cortical neurons to promote dose‐dependent silencing of huntingtin mRNA and protein expressions.^221^ This supports using exosomes in developing effective therapeutic strategies for HD.

Serum exosomes can be a valuable tool in treating HD.^222^ Transplantation of serum exosomes from mice into HD cell models in vitro effectively improved mHTT mutation and mitochondrial biogenesis and inhibited cell apoptosis. ADSC‐Exos release neurotrophic factors, which reduce mHTT aggregation and alleviate mitochondrial dysfunction and apoptosis.^223^, ^224^ αB‐crystallin is a small heat shock protein enriched in ADEs. It is involved in the secretion of exosomes to reduce the cytotoxicity of misfolded proteins and contribute to neuron survival. In HD astrocytes, mHTT reduced levels of αb‐crystallin and exosome secretion, leading to noncellular autonomic neurotoxicity in HD. When exosomes containing αB‐crystallin were injected into the striatum of HD mice, the accumulation of mHTT in the striatum was reduced.^225^ This approach may provide a breakthrough for HD treatment.

The active mutation of silent transcription factor leads to no more silence of HTT, resulting in transcriptional dysfunction. Many treatments attempt to restore normal silent transcription factor expression. However, when exosomes containing miR‐124 were injected into the striatum of R6/2 transgenic HD mice in an attempt to restore the normal expression of RE1‐silencing transcription factor and reduce Huntington protein activation, there was no significant improvement in HD‐like behavior, possibly due to the limited therapeutic effect of miR‐124 or the inadequate dosage in exosomes.^226^, ^227^ There is still a long way to go to effectively apply engineered exosomes to the treatment of HD.

### 3.8 Amyotrophic lateral sclerosis

Amyotrophic lateral sclerosis (ALS) is a fatal neurodegenerative disease that causes progressive muscle weakness, atrophy, and paralysis. Progressive ALS culminates in respiratory failure and death, resulting from damaged upper motor neurons from the cortex to the brainstem and lower motor neurons that project from the spinal cord to the muscles. ALS is characterized by progressive paralysis and motor neuron death, and the median survival is only 2–5 years. Most cases are sporadic and 5%–10% are familial ALS (fALS).^131^ The most common pathogenic genes for ALS include superoxide dismutase (SOD1), TAR DNA binding protein 43 (TARDBP/TDP43), chromosome 9 open reading frame 72 (C9orf72), and fusion protein (FUS).^228^, ^229^ ALS is also considered to be a protein disease, and its pathological proteins (SOD1, TDP‐43, and FUS) may accumulate and interfere with neuronal function, ultimately leading to cell death.^230^

SOD1 mutants are present in exosomes isolated from the ALS model,^231^ which is taken up by neighboring cells via endocytosis or released into the extracellular environment via exosomes. Their transmission is involved in ALS progression.^232^ It was also reported that astroglia‐derived exosomes efficiently transported SOD1 to spinal cord neurons and selectively induced motor neuron death.^233^ TDP‐43 is an RNA/DNA‐binding protein that has an aggregation tendency and cytotoxicity after hyperphosphorylation and ubiquitination.^234^ TDP‐43 levels are elevated in ALS brain exosomes and can also be secreted by exosomes from nerve cells and primary neurons. TDP‐43 protein spreads like a “prion” through the secretion and endocytosis of exosomes to stimulate the cytoplasmic accumulation of TDP‐43 in recipient cells.^235^ Exosomes play an essential role in TDP‐43 transport and clearance of TDP‐43. In addition, TDP‐43 level was positively correlated with ALS progression and can be used as a biomarker for diagnosis and progression of ALS.^236^ The sarcoma FUS is an RNA/DNA‐binding protein that is dysregulated in ALS and is associated with the most aggressive type in young adults.^229^ The CUEDC2 gene has a ubiquitin‐binding motif and regulates the ubiquitin‐proteasome pathway in CSF exosomes. It is considered a candidate disease biomarker for ALS.^230^ The C9orf72 gene has a GGGGCC repeat expansion in intron 1, which is the most common cause of fALS.^237^ RNA transcripts with repetitive sequences can accumulate repetitive RNAs into toxic dipeptide repeat proteins (DRPs), which disrupt normal cellular functions and lead to ALS. Exosomes can bind DRPs and deliver them to cortical neurons.^238^ Although the mechanism of DRPs release and cellular uptake remains to be investigated,^239^ the involvement of exosomes in the propagation of DRPs has been demonstrated.^236^, ^240^ Furthermore, miRNA dysfunction may be involved in the pathogenesis of ALS through TDP‐43.

miR‐27a‐3p is downregulated in serum exosomes of ALS patients. It stimulated myoblasts, promoted osteoblast mineralization through the Wnt signaling pathway, and participated in the progression of ALS.^230^ miR‐124‐3p is highly enriched in neuronal exosomes, and its expression in exosomes derived from spinal motor neurons in ALS patients is increased and is significantly correlated with ALS severity.^236^, ^241^, ^242^ This provides preliminary evidence that miR‐124‐3p can be used as an indicator of ALS progression. miR‐34a is involved in neuronal differentiation and neurogenesis. Its dysregulation leads to early neurodegeneration. miR‐34a and miR‐335 increased ROS production and impaired mitochondrial antioxidant function. Similarly, miR‐625‐3p targets lncRNA‐p21 and mediates neuroinflammation, oxidative stress, and apoptosis.^243^ Nucleolar complex protein 2 homologs (NOC2L) are overexpressed in CSF exosomes of patients with sporadic ALS. Novel INHAT (inhibitor of histone acetyltransferase) repressor (NIR) inhibits p53‐mediated gene transfer activation by blocking histone acetylation. Therefore, the inhibition of NIR increases p53‐dependent apoptosis. Nucleolar stress may play a role in the pathogenesis of sporadic ALS.^244^ Therefore, NIR can be used as a biomarker of homeostasis in ALS patients. These findings can provide valuable insights into the pathogenesis of ALS via specific pathways and facilitate the search for new therapeutic targets.

Protein dysfunction is present in ALS, and the best treatment is to restore protein homeostasis for proper protein folding.^245^, ^246^ S2RM stem cells were injected into the nervous system and spread through exosomes to restore protein homeostasis.^167^ This suggests that exosome contents can be protected in vivo before reaching the neural targets. Exosomes can stably transfer genes into primary neurons without immunogenicity, which can realize effective management of ALS.^247^ Glutamate is the major excitatory neurotransmitter in the CNS, and excessive or long‐term activation of glutamate receptors leads to neuron degeneration and death.^248^ Exosomes isolated from neurons were internalized into astrocytes to increase the levels of miR‐124a and glutamate transporter 1 (GLT1). The subsequent in vivo and exogenous delivery of miR‐124a by stereotaxic injection into SOD1‐G93A mice significantly prevented the pathological loss of GLT1.^249^, ^250^ This highlights the association of other exosome‐related biomarkers with inflammatory pathways and ALS.

The NSC (neural stem cell)‐34 cell line is a common in vitro model of ALS, which overexpresses the human SOD1‐G93A mutant protein and has reduced mitochondrial oxidative capacity. Stromal cell exosomes protected ALS‐mutated NSC‐34 cells from oxidative damage and increased cell viability.^251^ Mouse adipose stem cell (ASC) derived exosomes (ASC‐Exos) can repair the mitochondrial damage in NSC‐34 cells by regulating SOD1 aggregation, reducing oxidative damage, and restoring mitochondrial function.^252^ Exosomal proteins isolated from ALS mice negatively regulated cell adhesion and apoptosis and played a neuroprotective role.^253^ Repeated administration of ASC‐Exos in SOD1‐G93A transgenic ALS mice can improve motor performance, protect lumbar motor neurons, neuromuscular junctions, and muscles, and reduce glial cell activation.^254^, ^255^ This provides a new perspective for the application of ASC‐Exos in the treatment of ALS without the risks of immune rejection, genetic instability, and malignant transformation associated with the use of native or engineered stem cells.

Astrocytes are most abundant in the CNS and function to regulate neuron development. The miRNA expression profiles of astrocytes and exosomes in ALS models are different and not affected by the expression of mutant SOD1‐G93A.^256^ Thus, miRNA compatibility should be established for better ALS treatment strategies in the future.

### 3.9 Epilepsy

Epilepsy occurs when an abnormal discharge of neurons in the brain leads to transient dysfunction of the CNS.^257^ The presence of epilepsy in neurodegenerative diseases is increasingly recognized,^258^ and its incidence is still underestimated.

In recent years, several studies have established the relationship among exosomes, miRNAs, and epilepsy.^259^, ^260^ hsa‐miR‐184 in exosomes of patients with mesial temporal lobe epilepsy with hippocampal sclerosis (mTLE + HS) modulate immunity, inflammation, apoptosis, and exacerbate neuronal death after epileptic state.^261^ Its downregulation has predictive and diagnostic value for mTLE + HS. Expressions of miR‐194‐2 and miR‐15a in serum exosomes of epileptic patients were significantly downregulated.^262^ In a rat model of epilepsy, miR‐346 and miR‐331‐3p were significantly downregulated in exosomes from anterior brain cells,^263^ which may provide new targets for the pathogenesis and treatment of epilepsy. The expression of coagulation factor IX (F9) in serum exosomes of epileptic patients was higher than that of healthy controls, whereas thrombospondin 1 (TSP‐1) was lower than that of healthy controls. F9 is a vitamin K‐dependent plasma protein that has beneficial effects on synaptic plasticity. TSP‐1 is an adhesion glycoprotein secreted by astrocytes to promote synaptic development, neuronal migration, and axon growth.^264^ This is the first study of exosomal proteins in epilepsy, and the results have proven that proteins in exosomes may be a new tool for epilepsy diagnosis and treatment.

Status epilepticus (SE) can cause neuronal damage and glioma. Intranasal administration of BMSC‐Exos in epileptic mice improved neuronal loss, memory, and cognitive impairment. Treatment with BMSC‐Exos can also alleviate astrogliosis, inflammation, and mitochondrial dysfunction in LPS‐induced inflammation. Intraventricular injection of BMSC‐Exos in pilocarpine‐induced SE mice improved learning and memory impairment. Nrf2 (nuclear factor erythroid 2‐related factor 2) is an intermediary factor for neuroinflammation and oxidative stress. BMSC‐Exos can inhibit this factor, regulate the Nrf2‐NF‐κB signaling pathway, restore hippocampal astrocyte activation, and play an anti‐inflammatory role in treating epilepsy.^265^

### 3.10 Stroke

Stroke is an acute cerebrovascular disease, which is caused by the sudden rupture of blood vessels in the brain or blockage of blood circulation in the brain, resulting in focal neurological deficits. It is the second leading cause of death and disability in the world.^266^ The incidence of stroke is increasing due to an aging population. Cerebrovascular diseases are divided into hemorrhagic and ischemic, depending on their pathogenesis.^267^ Ischemic strokes (IS) are more common, but hemorrhagic strokes (HS) lead to higher rates of mortality and disability.

Circulating exosomal miR‐223 and miR‐134 were positively correlated with the occurrence, severity, and short‐term prognosis of IS.^124^ As biomarkers of IS, the value of exosomal miRNAs is higher than that of plasma miRNAs. The serum exosomal miR‐152‐3p level decreased in patients with IS, related to the degree of neurological deficit.^267^ As an indicator of nerve damage and neurotoxicity, serum exosome miR‐9 in patients with IS was associated with infarct volume, inflammatory status, and poor prognosis. Serum exosomal miR‐152‐3p is decreased in IS patients, which is related to the degree of neurological deficit.^268^ Large artery atherosclerosis (LAA) stroke is the most common type of IS. Analysis of exosomal miRNAs and LAA stroke revealed that miR‐369‐3p, miR‐493‐3p, miR‐379‐5p, and miR‐1296‐5p were differentially expressed. miR‐369‐3p is associated with low‐density lipoprotein and mononuclear macrophage differentiation, and its targeted genes are involved in the inflammatory process. miR‐493‐3p can regulate angiogenesis in IS rat model, and both miR‐493‐3p and miR‐379‐5p are involved in fatty acid metabolism and the pathogenesis of LAA. The expression levels of miR‐493‐3p and miR‐1296‐5p were negatively correlated with the National Institutes of Health Stroke Scale (NIHSS). miR‐1296‐5p is associated with cell adhesion molecules and plays a key role in atherosclerosis. miR‐369‐3p, miR‐493‐3p, miR‐379‐5p, and miR‐1296‐5p are promising biomarkers for the diagnosis of LAA stroke.^269^ Another study reported that lnc_000048, lnc_001350, and lnc_016442 in exosomes could effectively diagnose LAA stroke. lnc_001350 and lnc_016442 significantly improved the prediction of NIHSS in the prognosis of LAA stroke.^270^ Exosomal lncRNA can be a valuable biomarker for the prognosis of LAA stroke and change the outcome of LAA stroke.

Carotid plaque instability is an independent risk factor for IS. Circ_0043837 in plasma exosomes is related to mitochondrial function. Hence, mitochondrial damage affects the function of macrophages and participates in atherosclerosis. Circ_0001801 is involved in gene regulation and cellular protein modification. The expressions of both circ_0043837 and circ_0001801 are negatively correlated with NIHSS and stroke severity, prompting a protective effect on IS.^271^, ^272^ These biomarkers have better diagnostic effects than other plasma circRNAs in predicting atherosclerotic plaque rupture. Upregulation of circ_0006896 in serum exosomes promoted the migration and proliferation of human umbilical vein endothelial cells, resulting in carotid plaque instability. The downregulated expression of miR‐1264 leads to the increase of DNMT1 (DNA methyltransferase 1) and STAT3 phosphorylation levels, and the downregulation of SOCS3 (suppressors of cytokine signaling 3). The effects are reflected in the reduced inhibition of the JNK (c‐Jun N‐terminal kinase)/STAT3 pathway and the formation of vulnerable carotid plaques.^273^ The existence of the circ_0006896‐miR‐1264‐DNMT1 pathway in exosomes provides a new perspective for the study of carotid‐related IS. hsa_circ_0112036 and hsa_circ_0066867 in serum exosomes of IS patients regulated AMPK and PI3K‐AKT signaling pathways associated with IS pathogenesis. Upregulation of hsa‐circ_0093708 in plasma exosomes of IS patients increases the chance of brain injury by mediating hsa_circ_0093708‐miR‐4533‐AQP4 (aquaporin protein‐4) axis, and downregulating AQP4 can improve brain astrocyte injury. Upregulation of hsa_circ_0066867 and hsa_circ_0041685 regulated chemokine signaling through miR‐6737‐5p‐CCL2 and miR‐3192‐5p‐CXCL12, respectively, affecting leukocyte migration during inflammation and atherosclerosis development. Upregulation of hsa_circ_0041685 led to endocytosis and neuronal death and aggravated brain injury.^274^ The roles of exosomal ncRNAs, lncRNAs, and circRNAs in IS are not fully understood. Thus, it is feasible to further study them and improve the exosome‐based treatments for IS.

Injection of exosomes into aged ischemic rats reduced synaptic damage and improved the functional outcome of IS. The levels of proinflammatory mediators complement components 1Q (C1q), C3a, and C3b in serum exosomes increase with age. C3a receptors (C3aRs) regulate the degree of phagocytosis in microglia, which affects the prognosis of stroke. Selective microglial C3aR inhibitors can alleviate synaptic dysfunction and promote functional recovery after IS in elderly rats.^275^

Middle cerebral artery occlusion (MCAO) damages the structure and function of synapses, resulting in neurological dysfunction. Serum exosomes from the treatment group effectively inhibited microglia activation, increased the number of synapses and the expression of plasticity‐related proteins, reduced infarct volume, and improved neural function.^276^, ^277^ Thus, exosomes can provide a new therapeutic strategy for neuroprotection and repair after stroke. miR‐190b in ADEs inhibited apoptosis and increased neuronal survival under ischemic conditions.^278^ Exosome miR‐21 reduced TNF expression by inhibiting the NF‐κB pathway and increased the anti‐inflammatory factor IL‐10.^279^ It promoted the polarization of microglia cells toward the M2 phenotype.^280^ Macrophages are involved in the immune regulation of strokes, and exosomes secreted by macrophages also participate in the treatment of stroke. Administration of M2 macrophage exosomes after MCAO significantly inhibited glial scar formation and the activation and proliferation of astrocytes.

miR‐138‐5p carried by BMSC‐Exos targets lipocalin‐2 (LCN2) in astrocytes, which promotes cell proliferation and inhibits inflammation and apoptosis, thus reducing nerve injury after stroke.^281^ In addition, miR‐133b promotes functional recovery, whereas miR‐21‐5p promotes angiogenesis,^282^ both of which can be used in treating IS. In a rat model of intracerebral hemorrhage, BMSCs promoted neural function by transferring exosomal miR‐21 to neurons. miR‐193b attenuated brain edema and blood‐brain barrier injury.^191^ miR‐150‐3p is the most expressed exosomal miRNA, which has neuroprotective effects in vivo and in vitro. The expression of IS miR‐150‐5p was downregulated in BMSC‐Exos in MCAO rats and IS patients, leading to a poor prognosis and high mortality from IS. BMSC‐Exos improved nerve function and pathological changes in MCAO rats and reduced neuronal apoptosis and inflammatory factors.^283^ miR‐150‐5p protects cerebral ischemia–reperfusion (I/R) injury and provides a new therapeutic target for treatment. miR‐124 secreted from the M2 microglia can reduce astrocyte migration, proliferation, and scar formation by inhibiting STAT3 expression.^125^ Upregulation of exosomal miR‐124‐3p improved hypoxic–ischemic brain damage in neonatal rats by targeting TNF receptor–associated factor 6 (TRAF6).^284^, ^285^ Similarly, the upregulation of miR‐124‐3p in BMSC‐EVs was suggested as a therapeutic method for the targeted repair of damaged neurons after stroke. Caspases (CASP) 2, the most conserved cysteine‐rich proteases, are important in initiating apoptosis. Neural stem cell–derived exosomes (NSC‐Exo) inhibited neuronal apoptosis after brain injury by targeting CASP2 with miR‐150‐3p, reducing the infarct size in MCAO models, and playing a neuroprotective role. This could inform the development of new treatments for brain injury.^286^ BDNF was loaded into NSC‐Exos to construct the engineered BDNF‐HNSC‐Exos. In the MCAO rat model, BDNF‐HNSC‐Exos can inhibit microglial activation, promote endogenous NSC differentiation in neurons, inhibit the inflammatory response, create a suitable microenvironment for nerve regeneration, and alleviate NSC stress injury.^287^ Neuronal exosomes loaded with quercetin target ischemic brain tissue, improve neuronal survival, and reduce I/R damage by scavenging ROS production.^288^ Macrophage‐derived exosomes loaded with curcumin have an antioxidant capacity, which can target cerebral ischemic areas, reduce ROS accumulation, inhibit mitochondria‐mediated neuronal apoptosis, and positively affect I/R therapy.^289^

The content of exosomes and their contents also changes when IS occurs,^290^ and involved in the pathophysiological progression of IS, contribute to the diagnosis of the disease.^291^ Stem cell–derived exosomes play an important role in the treatment of stroke models. Therefore, engineered exosomes may be a potentially effective repair strategy for IS injury. However, there is a long way to go to further study on how to target the delivered exosomes to the responsible lesions.^292^ This is a research direction to improve the clinical value of exosomes.

## 4 CONCLUSIONS AND PROSPECTS

The occurrence of neuropsychiatric disorders is increasing day by day,^293^ but there are limitations in diagnosis,^294^, ^295^ limited means of treatment, and unsafe drugs.^296^, ^297^, ^298^ Naturally derived biological humoral exosomes possess specific biomolecules that can be used as a “signature” of parent cells to reflect changes in their contents under pathological conditions. The occurrence and pathophysiological mechanism of the disease can be understood by detecting its changing contents. Blood‐derived exosomes as liquid biopsy specimens have the potential to be a new biomarker for the diagnosis of neuropsychiatric disorders.^299^ Exosomes have the unique properties of being safe, low immunogenicity, biodegradable, and crossing the blood–brain barrier. The lipid bilayer escorts the loading and transport of bioactive molecules in exosomes, enables them to mediate the communication between brain cells stably, and crosstalk the physiological and pathological processes of the brain. In addition to that, exosomes can also be therapeutic carriers of drugs, RNAs, or proteins through direct loading, passive incubation, and active loading,^300^ which have great clinical application value in the treatment of neuropsychiatric disorders.

Despite the unique advantages of exosomes as engineering vectors, there are still challenges to their clinical application. Mass production, isolation, and drug loading techniques are key factors that limit the therapeutic potential of exosomes once they are introduced into the clinic.^301^, ^302^, ^303^ The loading efficiency of natural compounds was positively correlated with the molecular weight of exosomes. However, when molecular weight exceeds 1109 Da, exosomes coated with natural compounds cannot cross the blood–brain barrier, limiting therapeutic dose.^304^ How to ensure the high quality and effectiveness of isolated and modified exosomes needs relevant scientific research and technical support. Exosomes from different sources have different characteristics and specificities for different cells. In the future, selecting exosomes suitable for loading bioactive molecules or drugs will be essential according to cell types and how to accurately direct exosomes to target cells.^305^ Macrophages in mononuclear phagocytic system are responsible for the metabolic clearance of exosomes. Data from pharmacokinetic studies indicate that the residence time of exosomes in circulation affects the clinical transformation of therapeutic effect. Increasing the concentration of exosomes around target cells and allowing them to efficiently release bioactive molecules or drugs to achieve the expected therapeutic effect is an urgent problem that should be solved.^306^ However, exosomes have not yet been approved for clinical use.^307^, ^308^ Whether this involves medical ethics and which departments are required to supervise them is subject to the formulation of guidelines or expert consensus and the introduction of relevant policies.

Some bioactive molecules that have protective effects on the CNS have unstable chemical structures under physiological conditions, easy to be hydrolyzed, and low bioavailability, which limits their clinical application.^309^, ^310^, ^311^ The presence of the blood–brain barrier in turn blocks the delivery of bioactive molecules to the brain. Exosomes, as innovative drug carriers, can cross the blood–brain barrier, opening up the possibility for the treatment of neuropsychiatric disorders.^312^ Exosomes as innovative nanostructures,^313^ can “escort” bioactive molecules to be absorbed by neuron cells.^314^ Currently, nanoscavenger carrying therapeutic drugs with controllable release characteristics can target diseased brain tissue through intranasal drug delivery system, trigger autophagy and calcium‐dependent exosome secretion, and clear pathological proteins, providing strong neuroprotective potential.^315^ Or coating native immune cells with exosomes,^316^ makes it become a scavenger to clear immune activation, as a new treatment platform for neuropsychiatric disorders. Experimental research focusing on increasing the number of exosomes extracted, such as promoting exosome biogenesis by gene knockout or adding reagents to the medium,^317^, ^318^ is trying to break through the low yield bottleneck and promote the clinical application of exosome transformation. Protein is effectively packaged into exosomes for photo‐controlled release to target cells,^319^ and exosomes are used for bioimaging and disease therapy.^320^ All these have created more opportunities for the diagnosis and treatment of neuropsychiatric disorders.

Research on engineering exosomes of bioactive molecules is rising, and how to efficiently use exosomes for the diagnosis and treatment of neuropsychiatric diseases is ongoing. Given that exosomes are important carriers for the transmission of genetic information and bioactive molecules from cell to cell, they are involved in intercellular communication, disease progression, and therapy. It is believed that exosomes can be a powerful tool in diagnosing and treating neuropsychiatric diseases in the future.

## AUTHOR CONTRIBUTIONS

Qingying Si wrote and edited the manuscript. Linlin Wu conceived the manuscript. Deshui Pang and Pei Jiang provided significant assistance. All authors have read and approved the final manuscript.

## CONFLICT OF INTEREST STATEMENT

Authors declare no conflicts of interest in this work.

## ETHICS STATEMENT

Ethics approval was not needed for this study.
